# Combined diffusion‐relaxometry MRI to identify dysfunction in the human placenta

**DOI:** 10.1002/mrm.27733

**Published:** 2019-03-18

**Authors:** Paddy J. Slator, Jana Hutter, Marco Palombo, Laurence H. Jackson, Alison Ho, Eleftheria Panagiotaki, Lucy C. Chappell, Mary A. Rutherford, Joseph V. Hajnal, Daniel C. Alexander

**Affiliations:** ^1^ Centre for Medical Image Computing and Department of Computer Science University College London London United Kingdom; ^2^ Biomedical Engineering Department King’s College London London United Kingdom; ^3^ Centre for the Developing Brain King’s College London London United Kingdom; ^4^ Women’s Health Department King’s College London London United Kingdom

**Keywords:** diffusion, inverse Laplace transform, placenta, microstructure, multimodal MRI, relaxometry

## Abstract

**Purpose:**

A combined diffusion‐relaxometry MR acquisition and analysis pipeline for in vivo human placenta, which allows for exploration of coupling between ${\text{T}}_{2}^{*}$ and apparent diffusion coefficient (ADC) measurements in a sub 10‐minute scan time.

**Methods:**

We present a novel acquisition combining a diffusion prepared spin echo with subsequent gradient echoes. The placentas of 17 pregnant women were scanned in vivo, including both healthy controls and participants with various pregnancy complications. We estimate the joint ${\text{T}}_{2}^{*}$‐ADC spectra using an inverse Laplace transform.

**Results:**

${\text{T}}_{2}^{*}$‐ADC spectra demonstrate clear quantitative separation between normal and dysfunctional placentas.

**Conclusions:**

Combined ${\text{T}}_{2}^{*}$‐diffusivity MRI is promising for assessing fetal and maternal health during pregnancy. The ${\text{T}}_{2}^{*}$‐ADC spectrum potentially provides additional information on tissue microstructure, compared to measuring these two contrasts separately. The presented method is immediately applicable to the study of other organs.

## 1 INTRODUCTION

The placenta provides the vital link between mother and fetus during pregnancy. It is implicated in many major pregnancy complications, such as pre‐eclampsia (PE) and fetal growth restriction (FGR).^1^ PE affects 3–5% of pregnancies^2^ and is a major cause of maternal and perinatal mortality.^3^, ^4^ Late onset FGR, defined as that diagnosed after 32 weeks,^5^ affects 5–10% of pregnancies.^6^ It is strongly associated with stillbirth,^7^, ^8^ pre‐eclampsia,^9^ and late preterm birth.^10^ For all these disorders, it is likely that placental dysfunction occurs before the onset of symptoms. New techniques for imaging the placenta therefore have the potential to improve prediction, diagnosis, and monitoring of pregnancy complications.

Placental MRI is emerging as a technique with substantial promise to overcome some disadvantages of ultrasound. For example, ultrasound parameters of fetal wellbeing are imperfect for determining which fetuses have late‐onset FGR and are at greatest risk of adverse perinatal outcome, as opposed to those that are constitutionally small but healthy.^6^, ^11^ Assessing the placenta with MRI has the potential to make this distinction. Two MRI modalities that show great promise for assessing placental function are ${\text{T}}_{2}^{*}$ relaxometry—which has the potential to estimate oxygenation levels,^12^, ^13^ and diffusion MRI (dMRI)—which can estimate the microstructure and microcirculatory properties.^14^, ^15^, ^16^, ^17^

${\text{T}}_{2}^{*}$ relaxometry exploits the inherent sensitivity of the transverse relaxation time to the biochemical environment of tissue. In particular, the paramagnetic properties of hemoglobin mean that the ${\text{T}}_{2}^{*}$ time constant can be used as a proxy estimation of oxygenation.^18^ In placental studies, ${\text{T}}_{2}^{*}$ is generally lower in FGR cases.^19^, ^20^, ^21^, ^22^ A typical experiment acquires gradient echo data at several echo times (TE), either in separate or multi‐echo scans, and hence estimates the ${\text{T}}_{2}^{*}$ constant of the tissue. No diffusion weighting is typically applied to these scans. Applying diffusion gradients with different strengths (*b*‐value) and directions provides sensitivity to various microstructural length scales and orientations. These measurements are usually taken at a fixed TE. In the placenta, dMRI has shown promise for discrimination between normal pregnancies and FGR,^14^, ^15^, ^23^, ^24^, ^25^, ^26^ and early onset PE.^16^ However, despite the large number of placental ${\text{T}}_{2}^{*}$ and dMRI studies in the literature, no method has shown sufficient discrimination between healthy pregnancies and those with complications to be introduced into routine clinical practice. Methods which combine multiple distinct measurements may provide a way to overcome this. Supporting Information Table S1 summarizes ${\text{T}}_{2}^{*}$ and dMRI studies in the placenta to date.

${\text{T}}_{2}^{*}$ and dMRI‐derived measures are both influenced by the presence and composition of distinct tissue compartments (or *microenvironments*). Diffusion‐relaxometry MRI can simultaneously measure multiple MR contrasts; for example, by varying both TE and *b*‐value it is possible to probe the multidimensional ${\text{T}}_{2}$‐diffusivity (or ${\text{T}}_{2}^{*}$‐diffusivity) space. MR experiments dating back to the 1990s have simultaneously measured diffusivity and ${\text{T}}_{2}$ 27, 28, 29, 30, 31; such experiments are often categorized as diffusion‐relaxation correlation spectroscopy (DRCOSY).^32^ These acquisitions naturally pair with multidimensional analysis techniques which quantify multiple tissue parameters simultaneously, and therefore have great potential to yield fine‐grained information on tissue microstructure. Such analysis techniques have been recently applied to combined diffusion‐relaxometry experiments in the context of nuclear magnetic resonance (NMR) spectroscopy, improving the ability the distinguish different compartments.^33^, ^34^ Recent work applying these techniques to imaging has applications in the T1‐diffusivity,^35^ ${\text{T}}_{2}$‐diffusivity,^36^, ^37^ and T1–${\text{T}}_{2}$‐diffusivity^38^ domains. These studies have shown that combining diffusion with other MR contrasts leads to more specific quantification of microscopic tissue compartments. One recent study demonstrated combined ${\text{T}}_{2}$‐diffusivity in the placenta,^39^ with the aim to separate signals from fetal and maternal circulations.

A major disadvantage of previous diffusion‐relaxometry experiments are the very long scan times required when varying multiple contrast mechanisms, such as the TE and diffusion encoding. In this paper, we propose a combined acquisition and analysis technique which can estimate the ${\text{T}}_{2}^{*}$‐ADC spectrum within a clinically viable timeframe. We apply this novel method in the placenta, an organ where ${\text{T}}_{2}^{*}$ and ADC have both been shown to be informative. As well as demonstrating simultaneous estimation of ${\text{T}}_{2}^{*}$ and diffusivity parameters within a clinically viable time, we hypothesize that the joint ${\text{T}}_{2}^{*}$‐ADC spectrum will provide additional information compared to the individual measures.

## 2 METHODS

### 2.1 Acquisition: Integrated ${\text{T}}_{2}^{*}$‐Diffusion sampling

**Figure 1:**
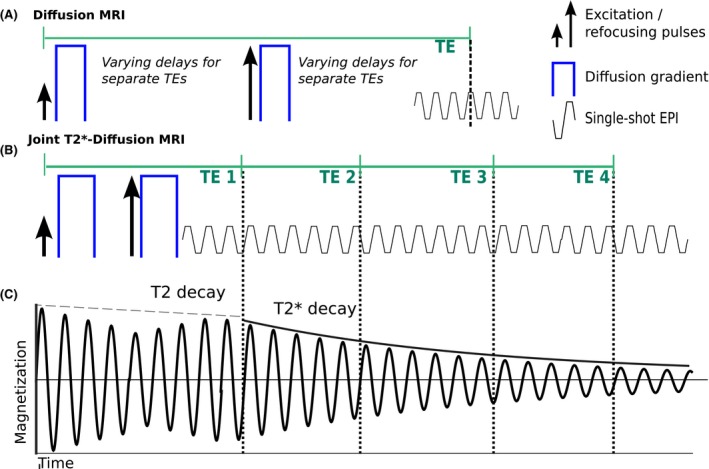
The considered acquisition schemes. A, Conventional Diffusion MRI acquisition for one echo time (TE) showing the diffusion gradients (blue), the excitation and refocusing pulses as well as the single‐shot EPI readout train. Repeating this acquisition with varying delays between the diffusion gradients and the readout leads to different TEs and thus combined ${\text{T}}_{2}$‐diffusion MRI. B, Proposed combined acquisition with an initial spin echo acquired after the diffusion gradients followed by multiple Gradient echos. C, Magnetization for the combined acquisition, with both ${\text{T}}_{2}$ and ${\text{T}}_{2}^{*}$ decay. The signal evolution neglects effects of all applied gradients

We adapt a novel MRI acquisition strategy, termed ZEBRA,^40^ in order to sample multiple TEs and diffusion encodings within a single repetition time (TR). The method combines a diffusion prepared spin echo sequence with subsequent gradient echoes. This allows simultaneous quantification of ${\text{T}}_{2}^{*}$ and ADC, as opposed to standard independent multi‐echo gradient echo and diffusion sequences (e.g. Figure 1A). Our technique also offers significant speed ups compared to existing ${\text{T}}_{2}$‐diffusivity techniques—which only sample a single TE‐diffusion encoding pair for each TR (i.e. Figure 1A). The proposed combined acquisition is shown in Figure 1B. The multiple gradient echoes are acquired with minimal spacing after the initial spin echo and diffusion preparation. We note that using gradient echo readouts rather than spin echoes, we measure ${\text{T}}_{2}^{*}$ rather than ${\text{T}}_{2}$ (see Figure 1C).

**Figure 2:**
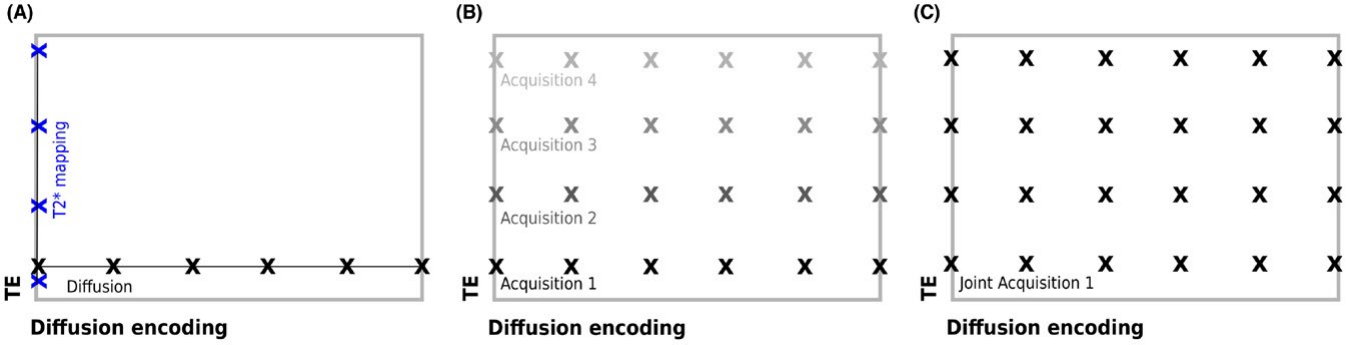
Schemes for the three considered diffusion‐relaxometry experiments illustrated in the TE‐diffusion encoding acquisition parameter plane. A, Schematic of conventional separate ${\text{T}}_{2}^{*}$ mapping and diffusion MRI showing the encoding of different echo times for *b* = 0 in blue and different diffusion encoding settings at fixed echo time. B, Parameter space illustrating the sampling of the TE‐diffusivity space with diffusion acquisitions at several TEs. Shading illustrates separate diffusion acquisitions at fixed TEs. C, Proposed combined ${\text{T}}_{2}^{*}$‐diffusion acquisition illustrating a denser sampling scheme achieved in a single acquisition

Figure 2 illustrates the resultant sampling of the TE‐diffusion encoding domain for the three acquisition techniques presented in Figure 1. Separate multi‐echo gradient echo and diffusion sequences do not adequately sample the full domain (Figure 2A). With repeat acquisitions of diffusion encodings at different TEs full sampling of the domain is possible, but very slow (Figure 2B). The proposed acquisition is able to sample the same domain in a much shorter, and clinically viable, scanning time (i.e. Figure 2C).

### 2.2 Modeling

The simplest model for analyzing the data considers single‐tissue compartments, so that the signal attenuations caused by ${\text{T}}_{2}^{*}$ relaxation and diffusion are both assumed to give rise to a single‐exponential decay. The MR signal for this combined ADC‐${\text{T}}_{2}^{*}$ model is given by

(1) $$S\left(T_{E},\;b\right)=S_{0}e^{-T_{E}/T_{2}^{*}}e^{-bADC}$$

where $T_{E}$ is the echo time, *b* is the *b*‐value, *ADC* is the apparent diffusion coefficient, ${\text{T}}_{2}^{*}$ is the effective transverse relaxation time, and $S_{0}$ is the signal at the spin echo time with zero diffusion weighting. $S_{0}$ is the product of proton density, ${\text{T}}_{2}$ weighting caused by finite spin echo time, receiver coil properties, and system gain, so we do not treat it as an absolute quantity in the analysis.

A shortcoming of this model is that it assumes the attenuation due to diffusion is monoexponential, when it is well established that the placental dMRI signal in vivo is at least biexponential, as in the intravoxel incoherent motion (IVIM) model.^41^ In this model, the slow and fast attenuating components are associated with diffusion in tissue and pseudo‐diffusion in capillaries, respectively. Incorporating ${\text{T}}_{2}^{*}$ decay into the IVIM model gives

(2) $$S\left(T_{E},\;b\right)=S_{0}e^{-T_{E}/T_{2}^{*}}\left[fe^{-bD^{*}}+\left(1-f\right)e^{-bADC}\right]$$

where *f* is the perfusion fraction and $D^{*}$ is the pseudo‐diffusion coefficient. However, it seems likely that the diffusion and pseudo‐diffusion compartments have different ${\text{T}}_{2}^{*}$ values. A model incorporating this was proposed by Jerome et al^42^

(3) $$S\left(T_{E},\;b\right)=S_{0}\left[fe^{-bD^{*}}e^{-T_{E}/T_{2p}^{*}}+\left(1-f\right)e^{-bADC}e^{T_{E}/T_{2}^{*}}\right]$$

where $T_{2p}^{*}$ and $T_{2}^{*}$ are the ${\text{T}}_{2}^{*}$ values specific to the pseudo‐diffusion and diffusion monoexponential signal components, respectively.

A significant limitation of the models presented in Equations (1)–(3) is that the number of signal components is assumed to be known. An alternative approach for analyzing the signal is a continuum model, which considers that spins have a spectrum of relaxivity (or diffusivity) values all contributing to the MRI signal. Following Menon et al^43^, the 1D continuum models for ${\text{T}}_{2}^{*}$ relaxometry and diffusion are

$$\begin{array}{cc} S(T_{E}) & =\int p\left(T_{2}^{*}\right)e^{-T_{E}/T_{2}^{*}}\;dT_{2}^{*}\\ S(b) & =S_{0}\int p\left(ADC\right)e^{-bADC}\;dADC. \end{array}$$

Here $p(T_{2}^{*})$ and *p*(*ADC*) are the ${\text{T}}_{2}^{*}$ relaxation and diffusivity spectra to be estimated from the data. We can solve for these spectra using an inverse Laplace transformation, although this is an ill‐posed problem requiring regularization to smooth the resulting spectra.^36^, ^38^, ^44^, ^45^, ^46^ The extension to combined diffusion‐relaxometry acquisitions is simple. For the acquisition presented here, where $T_{E}$ and *b* are simultaneously varied, the signal is (e.g.^47^)

(4) $$S\left(T_{E},\;b\right)=S_{0}\int_{0}^{\infty }p\left(T_{2}^{*},\;ADC\right)e^{-TE/T_{2}^{*}}e^{-bADC}\;dT_{2}^{*}dADC$$

The function we are interested in is the two‐dimensional ${\text{T}}_{2}^{*}$‐diffusivity spectrum, $p(T_{2}^{*},\;ADC)$, which can be estimated by a regularized 2D inverse Laplace transform. This contains more information than the individual 1D spectra, and is hence more likely to resolve multiple distinct tissue compartments. Although we emphasize that, due to choice of kernels in the continuum models, these distinct compartments—that is, separate peaks in 2D spectra—are assumed to be the result of monoexponential signal decays.

### 2.3 Experiments

The sequence described in the methods section was implemented on a clinical Philips Achieva‐Tx 3T scanner using the 32ch adult cardiac coil placed around the participant’s abdomen for signal reception. All methods were carried out in accordance with relevant guidelines and regulations; the study was approved by the Riverside Research Ethics Committee (REC 14/LO/1169) and informed written consent was obtained prior to imaging. Seventeen pregnant women, with gestational age ranging from 23+5 to 35+4 (weeks + days), were successfully scanned using the described technique. Three of these participants, one of whom also had FGR, were diagnosed with pre‐eclampsia according to standard definitions.^48^ Three participants had chronic hypertension in pregnancy and were analyzed distinct from normotensive pregnancy women (the control group). One pregnant woman with chronic hypertension was scanned twice, 4 weeks apart, and developed superimposed pre‐eclampsia by the second scan. The full participant details are given in Table 1.

**Table 1:** Participant details

Participant ID	GA at scan (weeks)	Cohort	TEs (ms)
1	23.72	Control	78, 114, 150, 186, 222
2	23.86	Control	78, 114, 150, 186, 222
3	25.43	Control	78, 114, 150, 186, 222
4	25.72	Control	78, 114, 150, 186, 222
5	26.14	Control	78, 114, 150, 186, 222
6	26.72	Control	78, 114, 150, 186
7	26.72	Control	78, 114, 150, 186, 222
8	27.14	Control	78, 114, 150, 186, 222
9	28.29	Control	78, 114, 150, 186, 222
10	28.86	Control	82, 175, 268, 361, 454
11	28.86	Control	78, 114, 150, 186, 222
12	29.67	Control	85, 145, 205, 265, 325
13	26.86	CH	80, 121, 162, 203, 245
14	34.43	CH	78, 114, 150, 186, 222
15	27.7	PE+FGR	78, 114, 150, 186, 222
16	30.58	PE	78, 114, 150
17 (scan 1)	30.71	CH	78, 114, 150, 186, 222
17 (scan 2)	34.14	CH+PE	78, 114, 150, 186, 222

Abbreviations: PE, pre‐eclampsia; CH, chronic hypertensive; FGR, fetal growth restriction.

The combined ${\text{T}}_{2}^{*}$‐diffusivity scan was acquired with the proposed sequence, a dMRI prepared spin echo followed by multiple gradient echos. The number and timing of the gradient echos varied across scans (see Table 1), with most scans having five TEs. The diffusion encodings were chosen specifically for the placenta, as previously reported,^49^, ^50^ with three diffusion gradient directions at *b* =  [5, 10, 25, 50, 100, 200, 400, 600, 1200, 1600] s mm${}^{-2}$, eight directions at *b* = 18 s mm${}^{-2}$, seven at *b* = 36 s mm${}^{-2}$, and 15 at *b* = 800 s mm${}^{-2}$. Further parameters were FOV = 300 × 320 × 84 mm, TR = 7 s, SENSE = 2.5, halfscan = 0.6, resolution = 3 mm${}^{3}$. One participant was scanned at higher resolution: 2 mm isotropic. The total acquisition time was 8 minutes 30 seconds. We acquired all images coronally to the mother. Attempting to acquire images in the same plane relative to the placenta would be very difficult, due to the heterogeneity in placental positioning and curvature across subjects. In clinical practice, the imaging plane with respect to the placenta has to vary widely; our samples allow us to demonstrate the method across a range of orientations. Supporting Information Figure S2 displays raw data from a single acquisition.

### 2.4 Model fitting

We first manually defined a region of interest (ROI) containing the whole placenta and adjacent uterine wall section on the first *b* = 0 image with the lowest TE. We fit the ${\text{T}}_{2}^{*}$‐ADC model described in Equation (1) voxelwise to the data (all TEs and all *b*‐values). The fitting consisted of two‐step (grid search followed by gradient descent) maximum log‐likelihood estimation assuming Rician noise, similar to that previously described,^17^ with the exception that we use the unnormalized MRI signal. The gradient descent fitting constraints were as follows: ${\text{T}}_{2}^{*}$ was constrained between 0.001 seconds and 1 second, the ADC between 10${}^{-5}$ and 1 mm${}^{2}$ s${}^{-1}$, and S0 between 0.001 and $10^{5}$. We fixed the SNR for fitting to 20 for all voxels in all scans.

**Figure 3:**
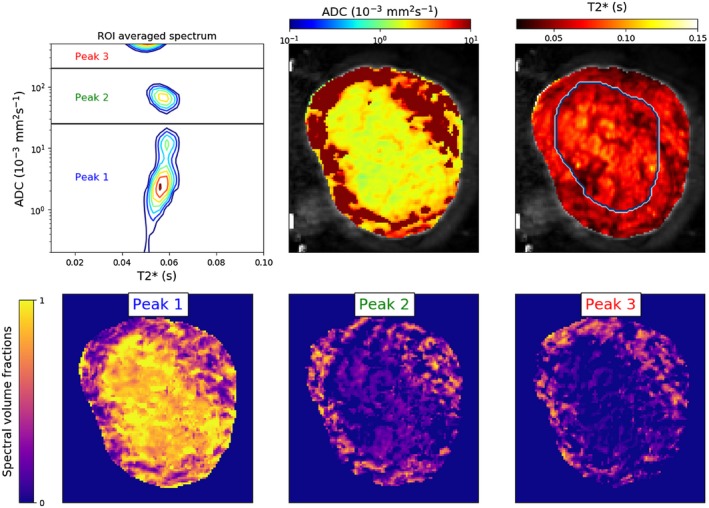
${\text{T}}_{2}^{*}$‐ADC spectra show anatomical specificity. Spatial maps for a single scan with higher resolution. Top row: ${\text{T}}_{2}^{*}$‐ADC spectrum derived from inverse Laplace transforms of the spatially averaged signal within an ROI comprising the entire placenta and uterine wall, and ADC and ${\text{T}}_{2}^{*}$ maps from combined ${\text{T}}_{2}^{*}$‐ADC fit. The manually defined placenta ROI is outlined in the ${\text{T}}_{2}^{*}$ map. Bottom row, spectral volume fraction maps derived by summing the weight of the spectra in the three domains displayed in the ROI averaged spectrum, as described in Methods

We calculated the ${\text{T}}_{2}^{*}$‐ADC spectrum for each participant from the signal averaged over the ROIs, using the MERA toolbox,^51^ which incorporates minimum amplitude energy regularization as described by Whittall et al.^52^ We also calculated the ${\text{T}}_{2}^{*}$‐ADC spectra voxelwise in all participants. We next quantified the spatial variation in ${\text{T}}_{2}^{*}$‐ADC spectral components across the placenta and uterine wall with volume fraction maps, using a similar approach to Benjamini et al^38^ and Kim et al.^36^ Specifically, by inspecting the ROI‐averaged spectra we chose a set of boundaries—based on the most common peak areas—which split the ${\text{T}}_{2}^{*}$‐ADC domain into regions. These boundaries were the same across all participants, and are given in Table 2. For each voxel’s ${\text{T}}_{2}^{*}$‐ADC spectrum, we then calculated the weight of the voxelwise spectra contained in each of these regions. By normalizing these weights to sum to 1 across all regions, we produced spectral volume fraction estimates for each voxel. Figure 3 shows an illustrative example of this calculation; the spectral volume fraction essentially quantifies the proportion of each voxel’s spectrum which lies in each of the highlighted regions in the top‐left panel.

**Table 2:** Boundaries selected to segregate most common peak areas in ${\text{T}}_{2}^{*}$‐ADC spectra

Region	ADC Bounds ($\times \;10^{-3}\;{\text{mm}}^{2}\;{\text{s}}^{-1}$)	${\text{T}}_{2}^{*}$ Bounds (s)
Peak 1	0 < *ADC* < 25	$0\;<\;T_{2}^{*}\;<\;0.1$
Peak 2	25 < *ADC* < 200	$0\;<\;T_{2}^{*}\;<\;0.1$
Peak 3	200 < *ADC* < 1000	$0\;<\;T_{2}^{*}\;<\;0.1$

## 3 RESULTS

**Figure 4:**
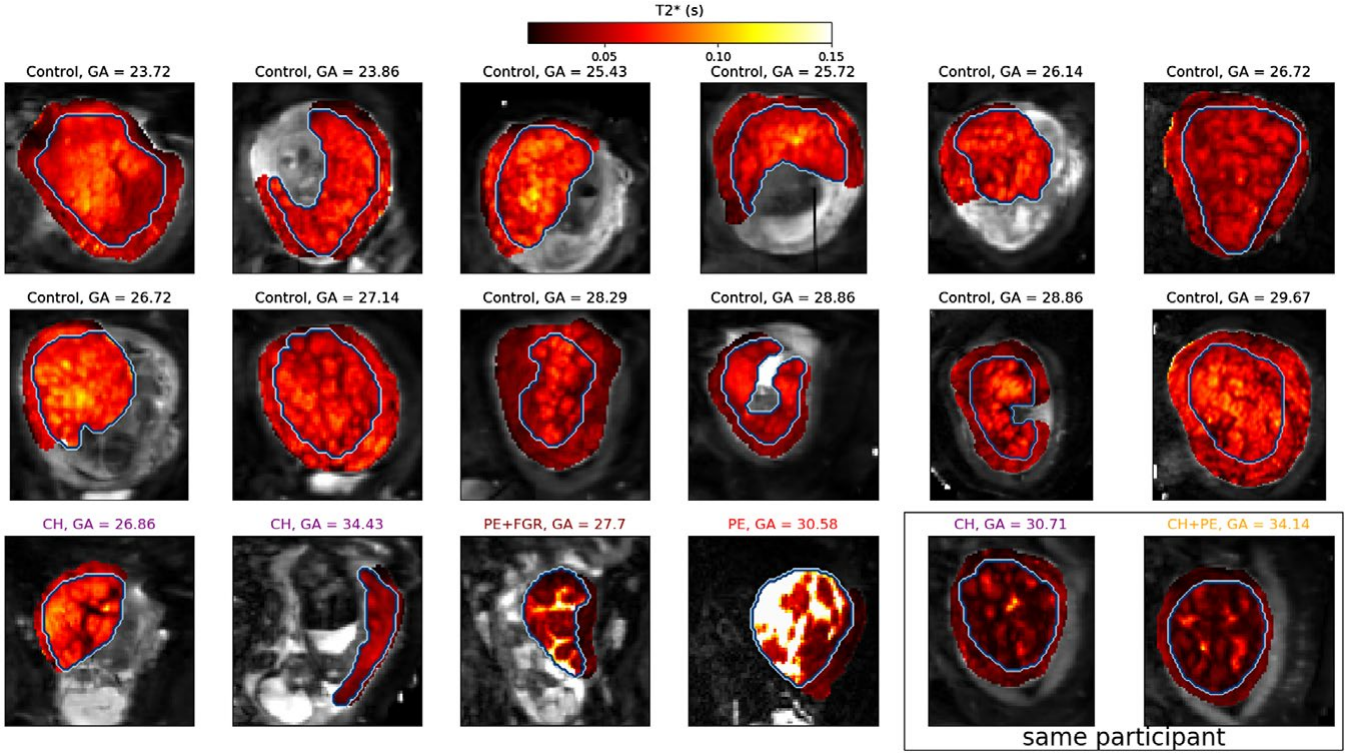
${\text{T}}_{2}^{*}$ maps from combined ADC‐${\text{T}}_{2}^{*}$ fit. Participants with pregnancy complications in color. The manually defined placenta ROI is outlined. Note the very high ${\text{T}}_{2}^{*}$ values for the GA = 30.58 participant—this is very likely due to model fitting failure caused by very low signal in this placenta

**Figure 5:**
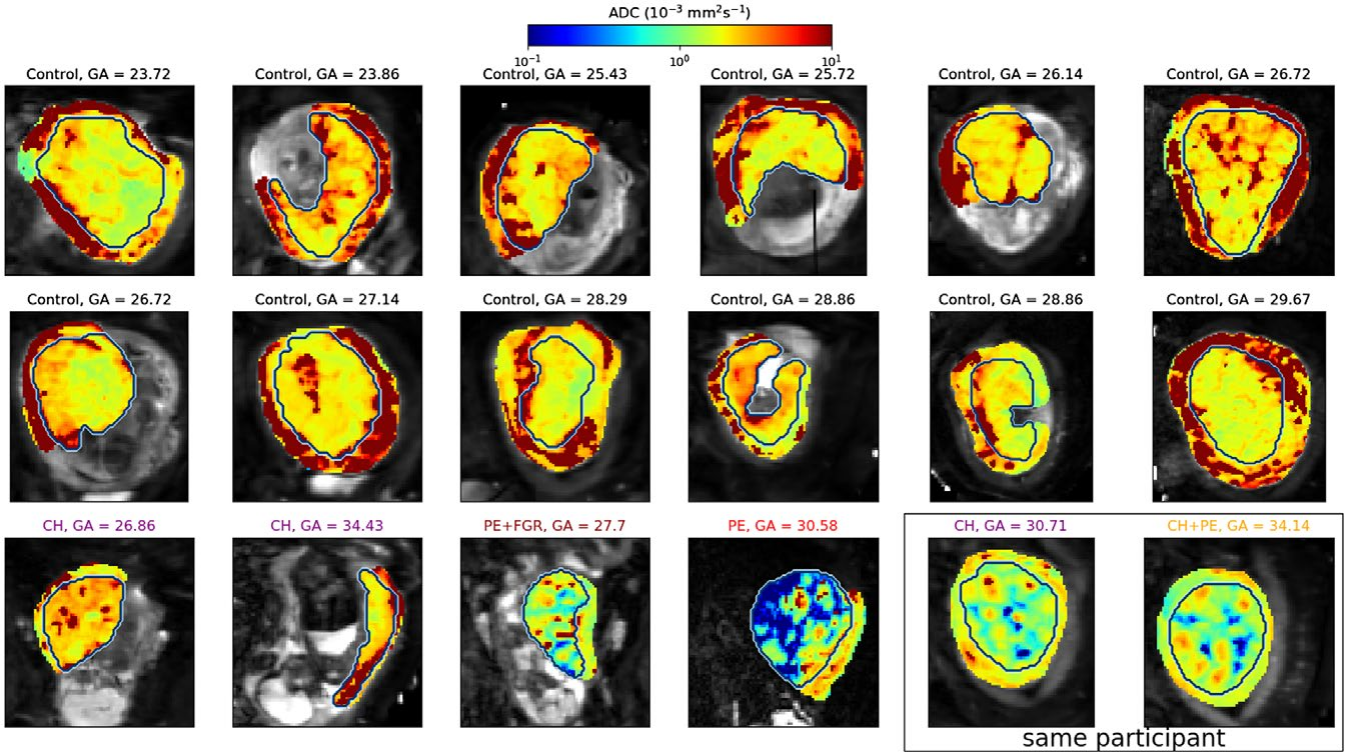
ADC maps from combined ADC‐${\text{T}}_{2}^{*}$ fit. The manually defined placenta ROI is outlined. Note the log‐scale colormap

**Figure 6:**
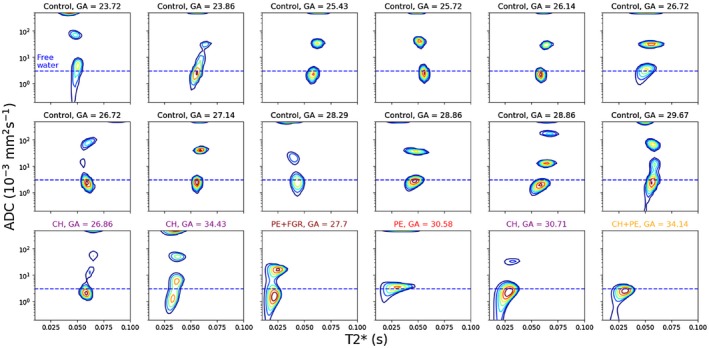
${\text{T}}_{2}^{*}$‐ADC spectra derived from inverse Laplace transforms of the spatially averaged signal within placenta and uterine wall ROIs. Horizontal dashed blue lines represent the approximate diffusivity of water in free media at $37^{\circ }$C ($3\;\times \;10^{-3}$ mm${}^{2}$ s${}^{-1}$)

**Figure 7:**
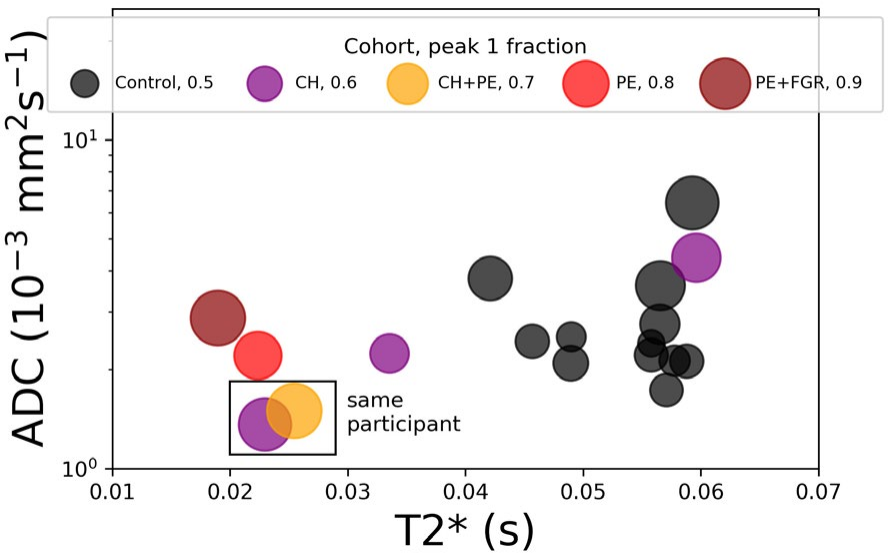
Position of the peak with the lowest ADC within the ADC‐${\text{T}}_{2}^{*}$ spectrum. Each marker corresponds to a single scan. Markers are colored by disease cohort, and marker area is proportional to the spectral volume fraction of the peak

Figure 3 demonstrates the full analysis pipeline output for a single participant. We next present the parameter maps from combined ADC‐${\text{T}}_{2}^{*}$ model fits (Figures 4 and 5) and spectral volume fraction maps (Supporting Information Figures S4‐S6) for all participants. We probe the changes across gestation and in disease cases by examining the ${\text{T}}_{2}^{*}$‐ADC spectra across all participants (Figures 6 and 7). Finally, in order to assess the independence of our diffusivity and relaxometry measurements, we plot the correlation between the derived ADC and ${\text{T}}_{2}^{*}$ values (Supporting Information Figure S6).

The first panel in Figure 3 shows the placenta and uterine wall ROI averaged ${\text{T}}_{2}^{*}$‐ADC spectrum for a single participant (scanned at higher resolution). We observe three peaks, clearly separated by ADC value but with similar ${\text{T}}_{2}^{*}$ values. ADC and ${\text{T}}_{2}^{*}$ maps show distinctive spatial patterns. The ADC is much higher in the uterine wall than the placenta. ${\text{T}}_{2}^{*}$ maps show distinct “lobes” surrounded by a patchwork of low ${\text{T}}_{2}^{*}$ values, with many lobes displaying a small region of higher ${\text{T}}_{2}^{*}$ in the center. The bottom row of Figure 3 displays voxelwise spectral volume fractions, obtained by integrating (i.e summing spectral weights) within three regions of the ${\text{T}}_{2}^{*}$‐ADC space, as described in Methods. The domain with the lowest ADC (e.g. peak 1) is associated with areas within the placenta, and the two domains (peaks 2 and 3) with higher ADC are more prominent in the uterine wall.

Figure 4 shows ${\text{T}}_{2}^{*}$ maps across all participants from the combined ${\text{T}}_{2}^{*}$‐ADC fit. The patterns are consistent with those previously reported in the literature.^50^, ^53^ In most participants regions of high ${\text{T}}_{2}^{*}$ encircled by low ${\text{T}}_{2}^{*}$ borders are clearly visible, and most likely correspond to placental lobules, with high ${\text{T}}_{2}^{*}$ indicating the presence of oxygenated blood. In agreement with previous observations, the regions with low ${\text{T}}_{2}^{*}$ are more prominent in pre‐eclampsia,^50^ and FGR^22^, ^54^ placentas.

ADC maps (Figure 5) also show anatomically linked qualitative features which are consistent across participants. In all scans from the healthy pregnant group the, ADC shows a significant increase at the border between the placenta and the uterine wall. This is most likely explained by the high levels of blood flow in these areas. This bordering area of high ADC is absent from many disease placentas. Additionally, placentas from women with chronic hypertension and pre‐eclampsia often show a distinctive pattern—small patches of high ADC surrounded by very low ADC.

Figure 6 displays the spatially averaged ${\text{T}}_{2}^{*}$‐ADC spectra for ROIs containing the placenta and uterine wall. We clearly observe separate peaks in all control participants, strongly suggesting the presence of multiple tissue compartments with distinct properties. In the vast majority (11/12) of these spectra from healthy controls, we see at least three clearly separated peaks. The ADC values of two of these peaks are typically above the diffusivity of water in free media (Figure 6, blue dashed lines), suggesting multiple microenvironments with different incoherent flow speeds. These peaks, and their corresponding tissue compartments, appear more clearly separated by ADC (note the log‐scale on the *y*‐axis) than by ${\text{T}}_{2}^{*}$ value. We also observed three distinct peaks in placentas from chronic hypertensive women. Interestingly, we did not see three distinct peaks in any spectra from participants with pregnancy complications (three PE, one PE+FGR). There is a distinct pattern in the ${\text{T}}_{2}^{*}$‐ADC spectra for the three PE participants—a left and downward shift in the lowest peak. This suggests a decrease in both ADC and ${\text{T}}_{2}^{*}$ distributions compared to control placentas. There is a similar leftward shift in the PE+FGR placental spectrum; however, the downward shift is not as pronounced, with the middle peak appearing to merge with the lowest peak. The peak with the highest ADC often appears to span the boundary of the domain in which the inverse Laplace transform is calculated. This is likely because we are unable to sample enough low *b*‐values to accurately estimate this very fast diffusing component—i.e. there is signal in the *b* = 0 volume, which has all attenuated by the *b* = 5 s mm${}^{-2}$ volume.

Spectral volume fraction maps showed similar patterns across all control participants (Supporting Information Figures S3‐S5); peaks with higher ADC being more prominent in the uterine wall. This likely reflects the high flowing blood volumes in these areas, akin to the maps in Figure 5.

Supporting Information Figure S6 shows that we did not observe a consistent correlation between ${\text{T}}_{2}^{*}$ and ADC values across participants. This suggests that we acquire complementary information from these two MR contrasts. Interestingly, we did not observe the small placental areas with high ${\text{T}}_{2}^{*}$ and high ADC that we saw in previous work.^50^

## 4 DISCUSSION AND CONCLUSION

### 4.1 Summary

This study demonstrates accelerated diffusion‐relaxometry MRI on the in vivo human placenta. Compared to existing approaches, it allows denser, faster, and more flexible sampling of the 2D (TE—diffusion encoding) acquisition space. This in turn allows visualization of the ${\text{T}}_{2}^{*}$‐ADC spectrum, and thus provides enhanced capacity to separate multiple tissue microenvironments. The technique was demonstrated on 17 pregnant participants, including 3 scans on placentas clinically assessed as from women with pregnancy complications. In the following sections, we first putatively associate the observed ${\text{T}}_{2}^{*}$‐diffusivity spectral peaks with distinct placental tissue microenvironments. We then hypothesize as to how the spectral changes observed in cases with complications reflect changes in these tissue microenvironments. Finally, we discuss the clinical potential of the presented technique, which we emphasize is independent of the biological interpretation.

### 4.2 Biological interpretation of ${\text{T}}_{2}^{*}$‐diffusivity spectra

In all controls, we observed a peak with high ADC, typically above $10^{-1}$ mm${}^{2}$ s${}^{-1}$. Additionally, in nearly every control participant (11/12) we observe two further clearly distinct peaks, with ADC around $2\;\times \;10^{-3}$ mm${}^{2}$ s${}^{-1}$ for the lower, and between $10^{-2}$ and $10^{-1}$ mm${}^{2}$ s${}^{-1}$ for the middle peak (Figure 6).

The appearance of three peaks clearly separated by diffusivity in all but one control placenta is consistent with each peak corresponding to a distinct placental tissue microdomain. Solomon et al. previously reported three placental compartments in mice,^55^ with these attributed to a slow‐diffusing maternal blood compartment, a fetal blood compartment with diffusivity around two orders of magnitude faster, and an intermediate compartment associated with active filtration of fluid across the fetal‐maternal barrier. We therefore speculatively assign tissue compartments to each of these three peaks in healthy control placentas as follows. The compartment with the lowest ADC, which has typical values ($2\;\times \;10^{-3}$ mm${}^{2}$ s${}^{-1}$) comparable to the diffusivity of water in tissue, is associated with water which is not subject to significant incoherent flow effects—this may be within tissue or slow‐moving maternal blood. The highest ADC compartment is associated with perfusing fetal blood, and the intermediate compartment with fluid transitioning between the maternal and fetal circulations—a significant proportion of which may reside within tissue. This is consistent with the spectral volume fraction maps for the peaks with higher ADC (Supporting Information Figures S4 and S5), which show higher intensity in the vascular areas bordering the placenta. The accuracy of these speculative tissue compartment assignments could be tested by comparison with ex vivo histology. Although such comparisons are notoriously challenging, achieving detailed correspondence would be highly valuable.

### 4.3 Spectral changes in disease

We observed three main trends in the ${\text{T}}_{2}^{*}$‐diffusivity spectrum which discriminated between control and placentas from women with pregnancy complications:

1. The disappearance of one (or both) of the middle and higher peaks
2. The lowest peak has a lower ${\text{T}}_{2}^{*}$
3. The lowest peak has a lower diffusivity

In placentas from women with pre‐eclampsia, we generally saw all three trends (Figure 6). The lower ${\text{T}}_{2}^{*}$ mirrors the previously reported decrease in ${\text{T}}_{2}^{*}$ in pre‐eclampsia placentas.^50^ We saw the same trend in the FGR+PE case, and note that lower ${\text{T}}_{2}^{*}$ values have also been observed in FGR placentas.^22^, ^56^ Regarding the lower diffusivity in the lowest peak, our initial speculation is that this could reflect increased water restriction due to inflammation—since placental inflammation is associated with PE.^57^ This may relate to the disappearance of the middle peak, which we hypothesis could reflect decreased maternal‐fetal fluid exchange. Inflammation is a potential mechanism facilitating the reduction in exchange, although we emphasize that this speculative link can only be confirmed (or refuted) by comparison with postdelivery histology. Figure 7 presents these observed changes in the ${\text{T}}_{2}^{*}$‐ADC spectrum in a single plot, showing clear separation between the control and pregnancy complication (i.e. PE, PE+FGR) participants. We plot the position of the spectral peak with the lowest ADC in the ${\text{T}}_{2}^{*}$‐ADC domain, with the marker area corresponding to the peak’s volume fraction. In this way, we capture both the peak shift, and the higher volume fraction due to the disappearance of the middle or higher peaks. Although these results are highly encouraging, we clearly need to scan many more participants, both control and women with pregnancy complications, to determine the discriminative power of these measures.

#### 4.3.1 Limitations and future work

We used an “out‐of‐the‐box” inverse Laplace transform toolbox to calculate the ${\text{T}}_{2}^{*}$‐ADC spectrum. There are a number of known weaknesses for this method, including the need for regularization. In this study, we chose minimum amplitude energy regularization. Future work could assess the utility of alternative optimization approaches, such as spatially constrained,^36^ or constrained by the 1D spectra.^38^

Our ${\text{T}}_{2}^{*}$ estimates are generally lower than those previously reported.^50^ This may be due to the larger voxel size, leading to partial volume effects around areas with high ${\text{T}}_{2}^{*}$, such as spiral artery inlets. It could also be due to signal attenuation due to diffusion during the gradient echoes, something which we did not account for in our analysis.

The presented ${\text{T}}_{2}^{*}$‐ADC spectral analysis assesses the data in two dimensions, but there are more dimensions to the data—such as diffusion gradient direction —which we did not include in our analysis. Therefore, this data set has the potential to be further analyzed, for example, with microstructural models that account for anisotropy in the signal.

In this study, we used *b*‐values and gradient directions optimized for dMRI at a single TE,^50^, ^58^ and the TEs were constrained by the EPI readout train length. Separate optimization of ${\text{T}}_{2}^{*}$ relaxometry and dMRI acquisition parameters is 1D (choice of TEs, choice of *b*‐values). However, when moving to combined ${\text{T}}_{2}^{*}$‐diffusion this becomes a 2D problem—for example, in the isotropic case we need to choose optimal TE‐diffusion encoding pairs. In future, we plan to optimize these TE‐diffusion encoding values in order to give the best sampling of the 2D parameter space, and enhance estimation of the 2D spectra.

### 4.4 Outlook and clinical application

The combined acquisition and analysis technique presented here offers the fast, simultaneous, and multidimensional assessment of placental ${\text{T}}_{2}^{*}$ and diffusivity in less than 10 minutes. These two MR contrasts have been shown elsewhere to be sensitive to placental pathologies, we hypothesize that their simultaneous assessment could enable better separation of healthy and poorly functioning placentas. This is supported by the fact that we did not see consistent correlation between ${\text{T}}_{2}^{*}$ and ADC values (Supporting Information Figure S6), suggesting that these modalities offer complementary information. This reinforces the value of the novel technique presented here as a quantitative tool for assessment of pregnancy complications, with the potential to ultimately inform clinical decisions. Furthermore, we believe that fast calculation of the ${\text{T}}_{2}^{*}$‐ADC spectrum has many potential applications in other areas of biomedical research.

## Supporting information


**TABLE S1** Overview of placental ${\text{T}}_{2}^{*}$ and dMRI studies to date
**FIGURE S1** Exemplary raw volumes from placental diffusivity‐relaxometry scan. The resolution was 2 mm isotropic—see Experiments section in Methods for further acquisition parameters. We display 70 out of the full set of 330 contrast encodings. Note that each row has a different color scaling. Figure 3 shows the derived ${\text{T}}_{2}^{*}$‐ADC spectrum and maps for this scan
**FIGURE S2** ${\text{T}}_{2}^{*}$‐ADC spectra derived from inverse Laplace transforms of the spatially averaged signal within placenta ROIs. Horizontal dashed blue lines represent the approximate diffusivity of water in free media at $37^{\circ }$C ($3\;\times \;10^{-3}$ mm${}^{2}$ s${}^{-1}$)
**FIGURE S3** Spectral volume fraction maps, obtained by summing the ${\text{T}}_{2}^{*}$‐ADC spectrum weight within the domain where $\mathrm{ADC}\;<\;25\;\times \;10^{-3}$ mm${}^{2}$ s${}^{-1}$
**FIGURE S4** As Figure S3, but for the domain where $25\;\times \;10^{-3}$ mm${}^{2}$ s${}^{-1}\;<\;\mathrm{ADC}\;<\;200\;\times \;10^{-3}$ mm${}^{2}$ s${}^{-1}$
**FIGURE S5** As Figure S3, but for the domain where $200\times 10^{-3}$ mm${}^{2}$ s${}^{-1}\;<\;\mathrm{ADC}\;<\;1000\;\times \;10^{-3}$ mm${}^{2}$ s${}^{-1}$
**FIGURE S6** Correlation between ${\text{T}}_{2}^{*}$ and ADC from combined ADC‐${\text{T}}_{2}^{*}$ fit within placental ROIs. Horizontal blue dashed lines represents the approximate diffusivity of water in free media at $37^{\circ }$C ($3\;\times \;10^{-3}$ mm${}^{2}$ s${}^{-1}$)
