# Supplementary material for: Combined diffusion‐relaxometry MRI to identify dysfunction in the human placenta
Source: Magn Reson Med. 2019 Mar 18;82(1):95–106. doi: 10.1002/mrm.27733 (PMC6519240; doi:10.1002/mrm.27733)
Supplement: Supplementary file 1 — TABLE S1 Overview of placental T2* and dMRI studies to date FIGURE S1 Exemplary raw volumes from placental diffusivity‐relaxometry scan. The resolution was 2 mm isotropic—see Experiments section in Methods for further acquisition parameters. We display 70 out of the full set of 330 contrast encodings. Note that each row has a different color scaling. Figure 3 shows the derived T2*‐ADC spectrum and maps for this scan FIGURE S2 T2*‐ADC spectra derived from inverse Laplace transforms of the spatially averaged signal within placenta ROIs. Horizontal dashed blue lines represent the approximate diffusivity of water in free media at 37∘C (3×10^-3^ mm2 s-1) FIGURE S3 Spectral volume fraction maps, obtained by summing the T2*‐ADC spectrum weight within the domain where ADC<25×10-3 mm2 s-1 FIGURE S4 As Figure S3, but for the domain where 25×10-3 mm2 s-1<ADC<200×10-3 mm2 s-1 FIGURE S5 As Figure S3, but for the domain where 200×10-3 mm2 s-1<ADC<1000×10-3 mm2 s-1 FIGURE S6 Correlation between T2* and ADC from combined ADC‐T2* fit within placental ROIs. Horizontal blue dashed lines represents the approximate diffusivity of water in free media at 37∘C (3×10^-3^ mm2 s-1) [file MRM-82-95-s001.pdf]

SUPPORTING INFORMATION TABLE S1 Overview of placental T2* and dMRI studies to date.

Reference	Parameters	Resolution	ROI selection
**T2***			
Sinding2016[56]	1.5T, gradient-recalled echo)	1.37x2.73x8mm	Entire placenta,
	16 TEs(3-67.5)	(2 slices, gap 2mm)	outer border not crossed
	BH 12s, 16 controls with repetitions		
Sinding2017[19]	1.5T, gradient-recalled echo)	1.37x2.73x8mm	Entire placenta,
	16 TEs(3-67.5)	3 slices	outer border not crossed
	BH 12s	transverse evenly	
Sinding2018[22]	1.5T, gradient-recalled echo)	1.37x2.73x8mm	entire placenta
	16 TEs(3-67.5)	3 planes evenly	adjusted for movements
	BH 12s, 16 HC with repetitions		
Derwig2013a[21]	1.5T, flow-compensated SE (ind. scans)	3.76x3.75x8	representative area of central part
	TEs= 40,80,120,180,240,300,360,440	3 slices, no gap	away from vessels
Ingram2017 [20]	gradient-recalled echo	3.52x3.52	largest contiguous placental region
	5-50ms, 8 sec BH, under O_2	1 slices transverse	non-placental tissue removed
Hutter2018[50]	2D ss EPI Multi-echo GE	2x2x2	conservative
**dMRI**			
Moore2000a[59]	0.5T, 11 b-values (0-468 s mm^{-2})	3.5×2.5×7 mm	Entire placenta
Moore2000b[14]	0.5T,11 b-values (0-468 s mm^{-2})	3.5×2.5×7 mm	Entire placenta
Derwig2013b[15]	1.5T, 11 b-values (0-500 s mm^{-2})	3.75×3.75×4 mm	Two: central, whole
Sohlberg2015[25]	1.5T, 5 b-values (0-800 s mm^{-2})	??×??×6 mm	excluding artefactual signal loss areas
You2017[60]	1.5T, 9 b-values (0-900 s mm^{-2})	4.38×4.38×4 mm	Entire placenta
Capuani2017[61]	1.5T, 7 b-values (0-1000 s mm^{-2})	2×2×4 mm	Three: central, peripheral, umbilical
Siauve2017[62]	1.5T, 11 b-values (0-1000 s mm^{-2})	??×??×5 mm	Three: entire placenta, fetal, maternal
Slator2017[17]	3T, 12 b-values (0-2000 s mm^{-2})	2×2×2 mm	Entire placenta
Jakab2017[63]	1.5T and 3T, 17 b-values (0-900 s mm^{-2})	2×2×4 mm	Central
Hutter2018[50]	3T, 14 b-values (0-1600 s mm^{-2})	2×2×2 mm	Entire placenta

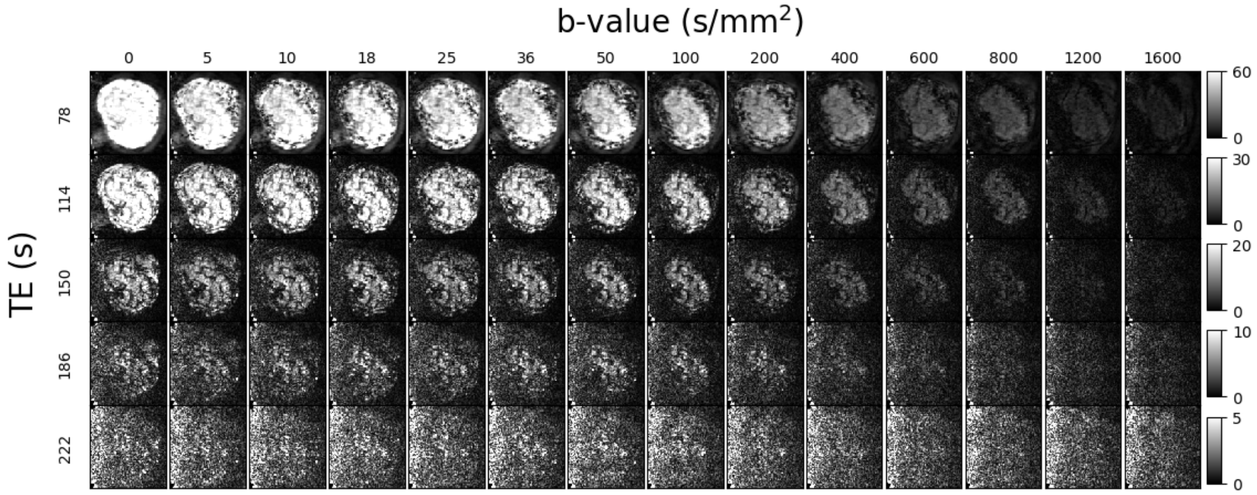

SUPPORTING INFORMATION FIGURE S1 Exemplary raw volumes from placental diffusivity-relaxometry scan. The resolution was 2 mm isotropic - see Experiments section in Methods for further acquisition parameters. We display 70 out of the full set of 330 contrast encodings. Note that each row has a different color scaling. Figure 3 shows the derived T2*-ADC spectrum and maps for this scan.

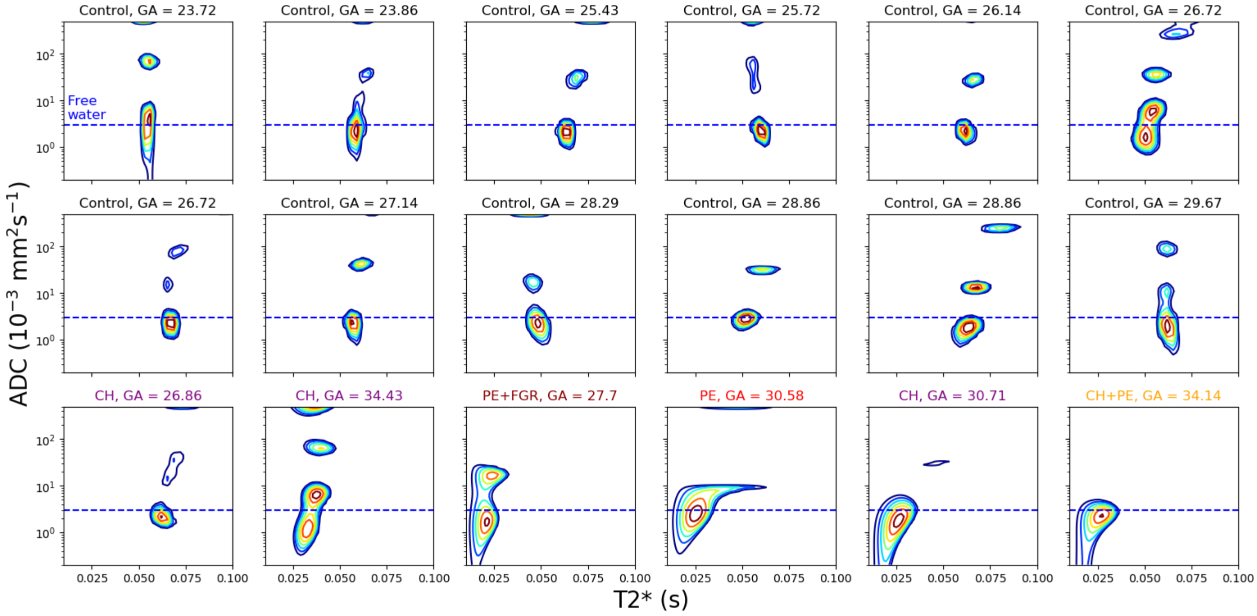

SUPPORTING INFORMATION FIGURE S2 T2*-ADC spectra derived from inverse Laplace transforms of the spatially averaged signal within placenta ROIs. Horizontal dashed blue lines represent the approximate diffusivity of water in free media at 37°C (3×10^{-3} mm^2 s^{-1}).

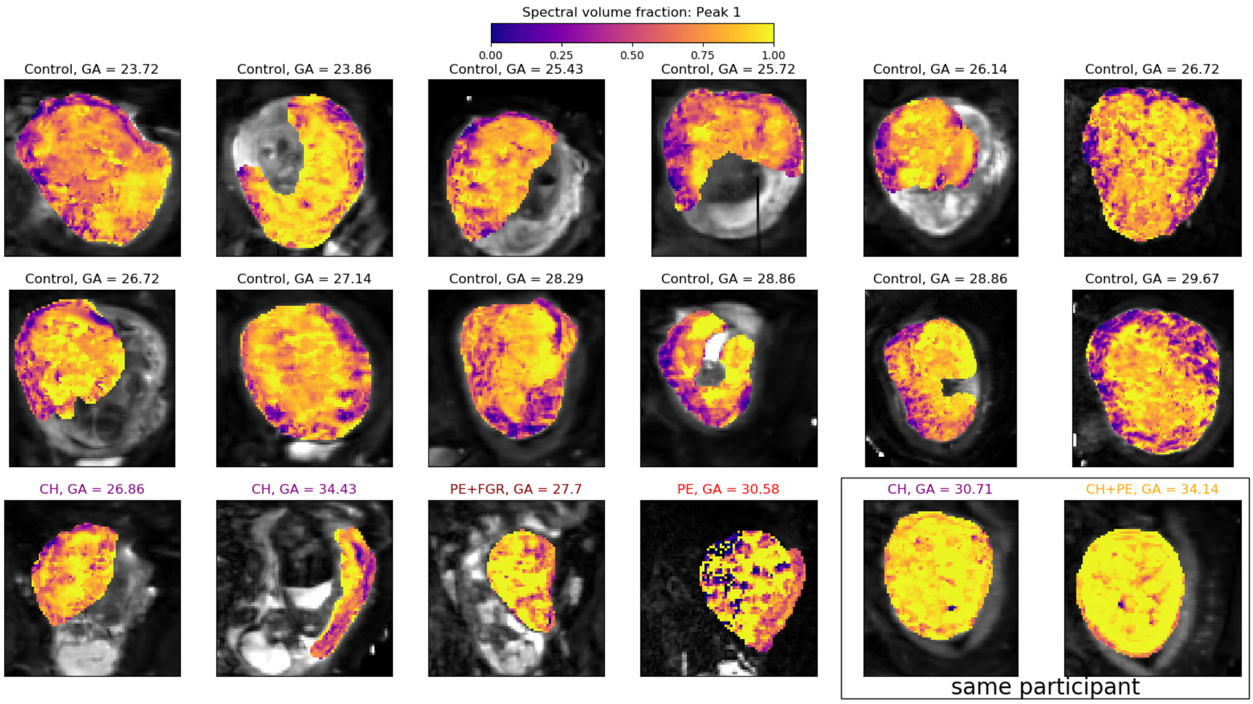

SUPPORTING INFORMATION FIGURE S3 Spectral volume fraction maps, obtained by summing the T2*-ADC spectrum weight within the domain where ADC $< 25 \times 10^{-3}$ mm^2 s^{-1}.

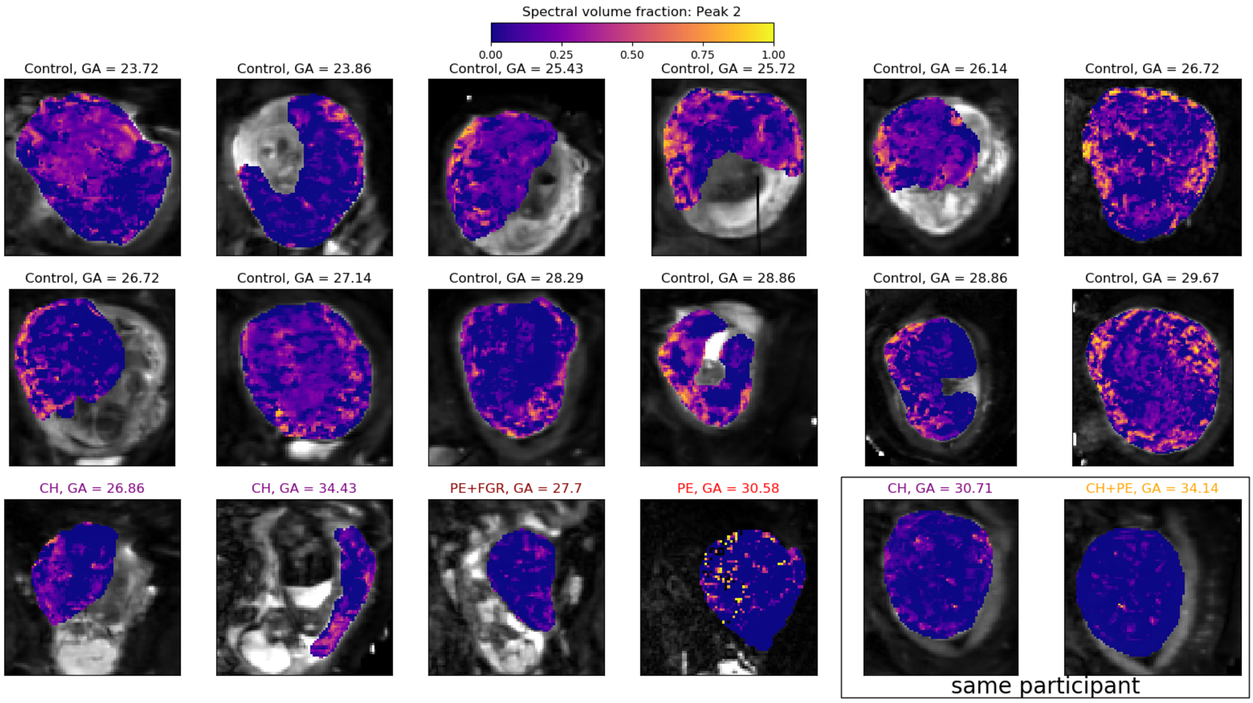

SUPPORTING INFORMATION FIGURE S4 As Figure S3, but for the domain where 25×10^{-3} mm^2 s^{-1} < ADC $< 200 \times 10^{-3}$ mm^2 s^{-1}.

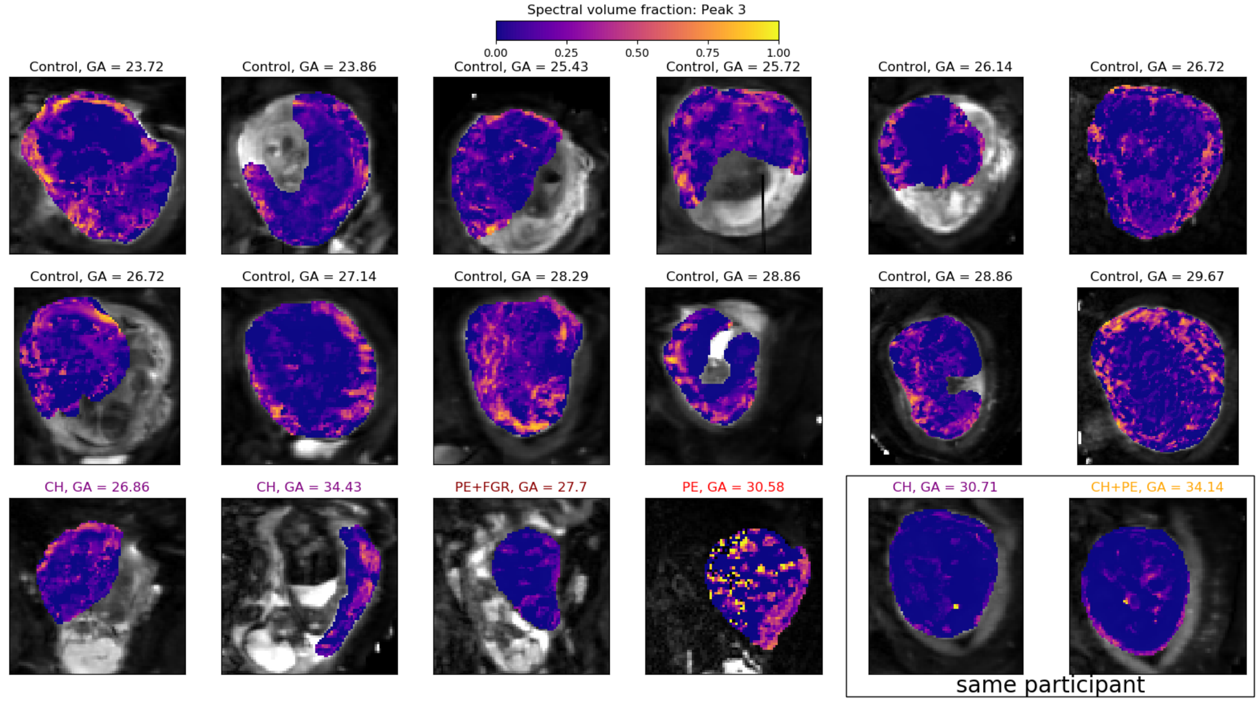

SUPPORTING INFORMATION FIGURE S5 As Figure S3, but for the domain where 200×10^{-3} mm^2 s^{-1} $<$ ADC $<$ 1000×10^{-3} mm^2 s^{-1}.

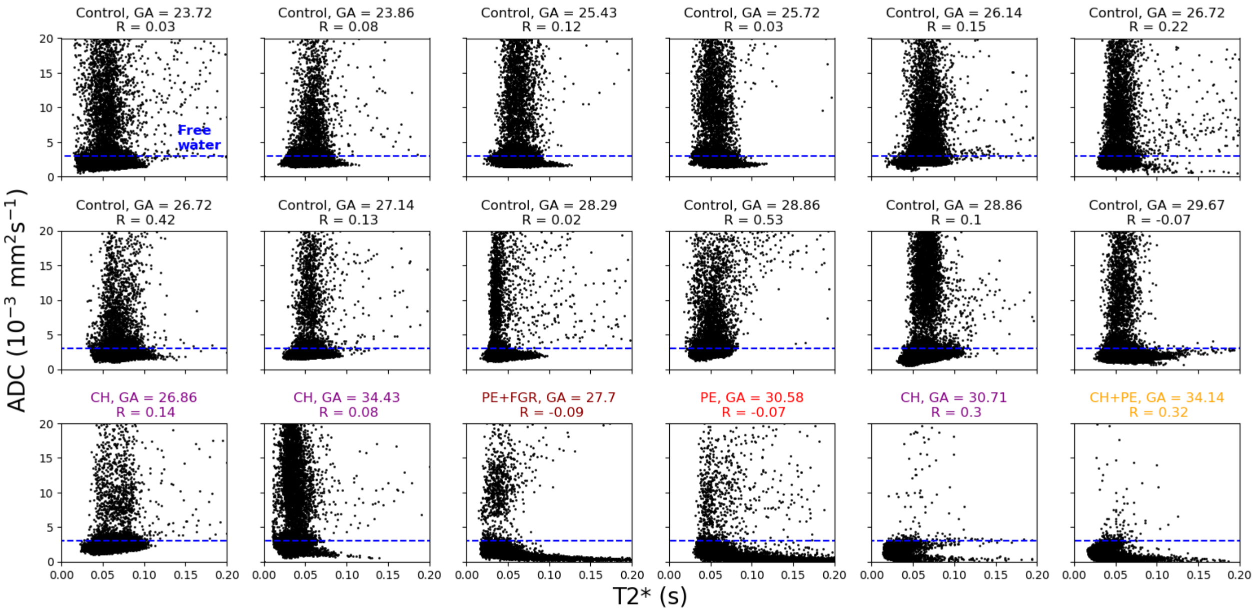

SUPPORTING INFORMATION FIGURE S6 Correlation between T2* and ADC from combined ADC-T2* fit within placental ROIs. Horizontal blue dashed lines represents the approximate diffusivity of water in free media at 37°C (3×10^{-3} mm^2 s^{-1}).
